# A randomized controlled comparative study of different fluid exchange modes in urgent-start peritoneal dialysis in patients with end-stage renal disease: automated peritoneal dialysis combined with manual fluid exchange vs. manual fluid exchange alone

**DOI:** 10.1080/0886022X.2023.2202756

**Published:** 2023-06-14

**Authors:** Xiaoxiao Xia, Xueqin He, Li Pu, Xia Liu, Xueli Zhou, Xiao Fang Wu, Zhiyun Zang, Zi Li

**Affiliations:** a Department of Nephrology, Sichuan University, West China Hospital, Chengdu, P.R. China; b West China School of Nursing, West China Hospital of Sichuan University, Chengdu, P.R. China; c Department of Nephrology, The Third Affiliated Hospital of Chongqing Medical University (Gener Hospital), Chongqing, P.R. China

**Keywords:** Automated peritoneal dialysis, manual fluid exchange peritoneal dialysis, urgent-start peritoneal dialysis, end-stage renal disease

## Abstract

During urgent-start peritoneal dialysis (USPD) in end-stage renal disease (ESRD) patients, both adequate dialysis and skill training for fluid exchange are essential. However, automated peritoneal dialysis (APD) alone or manual fluid exchange peritoneal dialysis (MPD) alone could meet the above demands. Therefore, our study combined APD with MPD (A-MPD), and compared A-MPD with MPD, aiming to find the most appropriate treatment mode. This was a single-center, prospective, randomized controlled study. All eligible patients were randomized into the MPD and A-MPD groups. All patients underwent a five-day USPD treatment 48 h after catheter implantation, and they were followed up for six months after discharge. Overall, 74 patients were enrolled in this study. Among these, 14 and 60 patients quit due to complications during USPD and completed the study (A-MPD = 31, MPD = 29), respectively. Compared with MPD, the A-MPD treatment mode had a better effect on removing serum creatinine, blood urea nitrogen, and potassium and improving serum carbon dioxide combining power levels; it had less time expenditure on the fluid exchange by nurses (*p* < 0.05). In addition, patients in the A-MPD group had higher scores on the skill tests than those in the MPD group (*p* = 0.002). However, no significant differences in short-term peritoneal dialysis (PD) complications, PD technical survival rate, or mortality were found between the two groups. Therefore, the A-MPD mode could be recommended as an adoptable and suitable PD modality for USPD in the future.

## 1. Introduction

Peritoneal dialysis (PD) is an effective treatment for patients with end-stage renal disease (ESRD). PD initiation is usually recommended at least two weeks after peritoneal dialysis catheter (PDC) implantation [1]. However, more than 40% of patients with ESRD require urgent initiation of dialysis on admission because of their unwillingness to accept renal replacement therapy, unexpected deterioration of renal function, and late referral to a nephrologist [2]. Therefore, in this setting, hemodialysis (HD) *via* a temporary venous catheter is the major modality that may also increase the incidence of catheter-related infections and central venous stenosis [3,4]. Recently, PD has been increasingly used for the urgent initiation of dialysis.

Urgent-start peritoneal dialysis (USPD) is defined as the initiation of PD within two weeks of PDC implantation. Interestingly, our previous meta-analysis showed that all-cause mortality was comparable between patients undergoing USPD and urgent-start HD; however, the incidence of bacteremia was higher in patients receiving urgent-start HD [5]. Therefore, USPD may be a feasible option for patients with ESRD who require urgent dialysis.

PD is usually performed using a manual fluid exchange peritoneal dialysis (MPD) or automated peritoneal dialysis (APD). APD has been proven to be a safe and effective alternative to urgent-start HD [6,7]. APD has recently been widely used in USPD because of its better effect in removing toxic and excess water than the traditional MPD, therefore, enabling patients to get through the transition period more easily [8,9]. However, unlike MPD, the APD mode does not provide skill training. Patients need to choose the PD mode according to their lifestyle and willingness to participate in their own care after discharge. Meanwhile, PD nurses usually use MPD mode to observe the flow and color of PD fluid for the first few bags after PDC implantation. Therefore, APD cannot be the only PD model in USPD.

Both adequate dialysis and skill training for fluid exchange are essential in USPD. However, APD alone or MPD alone could not meet the above demands. We hypothesize that a combination of APD with MPD (A-MPD) in USPD could have a better effect in removing small-molecule toxins and skill training. It may also allow the patients to receive multiple PD modes and choose the most suitable one for them. In this randomized controlled study, we compared A-MPD with MPD, aiming to investigate the efficacy and practicability of A-MPD and seek the most appropriate mode in ESRD patients undergoing USPD.

## 2. Materials and methods

### 2.1. Design

This single-center, prospective, randomized, open-label, controlled study was approved by the Ethics Committee of West China Hospital of Sichuan University (approval number: 2020-891) and registered in the Chinese Clinical Trials Registry (www.chictr.org.cn, registration number: ChiCTR2000038620) before the first patient was enrolled. The study was conducted following the Declaration of Helsinki, and written informed consent was obtained from the participants.

### 2.2. Participants

Nephrologists provided patients with ESRD requiring urgent dialysis with detailed information on possible treatment modalities. Individuals who preferred PD and were suitable for PD were eligible if they were aged 18–75 years. The study recruitment period was from March to December 2021 at the Department of Nephrology, West China Hospital of Sichuan University.

Exclusion criteria were as follows: (1) conditions requiring emergent hemodialysis, including uncontrolled severe acute heart failure (New York Heart Association, Grade IV), uremic encephalopathy, severe electrolyte disorders (serum potassium [s-K+] > 6.5 mmol/L), and unstable vital signs; (2) complications associated with other serious diseases, such as severe systemic infections or sepsis; and (3) refusal to participate in the study; and (4) participation in other clinical studies.

All eligible patients were randomized into two groups according to a computer-generated randomization chart; the MPD and A-MPD groups. In addition, general information was recorded, including age, sex, causes of ESRD, body mass index (BMI), body surface area (BSA), surgical PDC implantation method, and laboratory measurements.

### 2.3. PDC implantation and PD schemes

All patients received Tenckhoff catheters and prophylactic antibiotics were administered preoperatively. Two experienced nephrologists performed catheter implantations using open dissection, and those through laparoscopy were performed by a surgeon. All the PDC were implanted at the day surgery center. USPD was initiated 48 h after PDC implantation and continued for five days during hospitalization.

The PD schemes were based on the patients’ BSA [10] and previous studies [8–12]. Subsequently, the patients in the MPD group underwent four exchanges with a low intraperitoneal volume of 700 mL and a dwell time per exchange of 180 min when the BSA was <1.7 m^2^. Patients in the A-MPD group underwent the MPD regimen (two exchanges of 700 mL for 180 min per session) and the APD (PDGO Automated Peritoneal Dialysis cycler, Fuzhou Dongze Medical Device Co., Ltd, China) regimen (tidal PD mode for 600 min of treatment in the supine position with an initial dose of 700 mL; the cyclers delivered five cycles of 500 mL dialysate with a dwell time of 100 min per session). When BSA was >1.7 m^2^, the intraperitoneal volume increased accordingly. Table 1 presents the details of the PD scheme.

**Table 1.** Standard prescription for the patients of USPD.

Group	BSA	Schemes /day	Curative time /day	Total dialysate volume /day
A-MPD	<1.7m^2^	M (700 mL × 2 exchanges) + A (3200 mL/ 600 min/ 5 cycles)	960 min	4600mL
	≥1.7m^2^	M (1000 mL× 2 exchanges) + A (5000 mL/ 600 min/ 5 cycles)	960 min	7000mL
MPD	<1.7m^2^	700mL × 4 exchanges	720 min	2800mL
	≥1.7m^2^	1000mL × 4 exchanges	720 min	4000mL

A-MPD: automated peritoneal dialysis combined with manual fluid exchange peritoneal dialysis; MPD: manual fluid exchange peritoneal dialysis.

Nurses treating PD taught and assisted patients in completing manual fluid exchanges and performing the cyclers. All patients were trained in manual fluid exchange by nurses treating PD during hospitalization and were administered the test before discharge. However, patients who failed the test were retrained and reevaluated until they qualified. The time spent by the nurses treating PD and the test scores of the patients were recorded. Finally, all patients were followed up six months after discharge.

### 2.4. Termination of the study

When patients experienced complications, such as leakage and mechanical complications of PDC, which disturbed normal PD therapy, medication and conservative treatment were administered, and PD was suspended for 3 days. In addition, the patients continued the study and completed the five-day USPD after such complications were resolved. Otherwise, the patients would terminate the study, and further treatment would involve replacing the PDC under laparoscopy, suspension of PD for a longer time, or initiating urgent hemodialysis if needed. The patients also terminated the study once peritonitis or inguinal hernia occurred.

### 2.5. Outcome measurements

The primary outcomes included clearance of small-molecule uremic toxins and PD technical survival rate. All patients underwent laboratory measurements before and after USPD, including serum levels of creatinine, blood urea nitrogen, albumin, sodium, potassium, magnesium, phosphorus, carbon dioxide combining power (CO_2_-CP), intact parathyroid hormone (iPTH), hemoglobin, and N-terminal pro-brain natriuretic peptide (NT-pro-BNP). Self-comparison of uremic toxins (serum creatinine, blood urea nitrogen, potassium, magnesium, phosphorus, CO_2_-CP, and iPTH) was conducted within the group. The differences in these measurements before and after USPD were calculated and compared between the two groups. PD technical survival was defined as the patient being alive at the end of follow-up and still undergoing PD.

Secondary outcomes included the incidence of short-term PD-related complications (including peritoneal fluid leakage, PDC malfunction, peritonitis, catheter-related infection, and inguinal hernia), time expenditure of nurses treating PD, and patient test scores. Catheter-related infections included exit-site infection and tunnel infection [13].

### 2.6. Statistical analysis

All statistical analyses were performed using the Statistical Package for Social Sciences (SPSS) software (version 26.0). Continuous variables, which conformed to the normal distribution, were expressed as mean ± standard deviation, and comparisons between groups were performed using the Student’s *t*-test. In contrast, continuous variables that conformed to the non-normal distribution were expressed as medians or quartiles, and non-parametric tests were performed to compare groups. Categorical variables were described as frequencies or percentages, and comparisons between groups were performed using the *χ^2^* test. Statistical significance was considered at *p* < 0.05.

## 3. Results

### 3.1. Demographic and baseline characteristics

**Figure 1.**
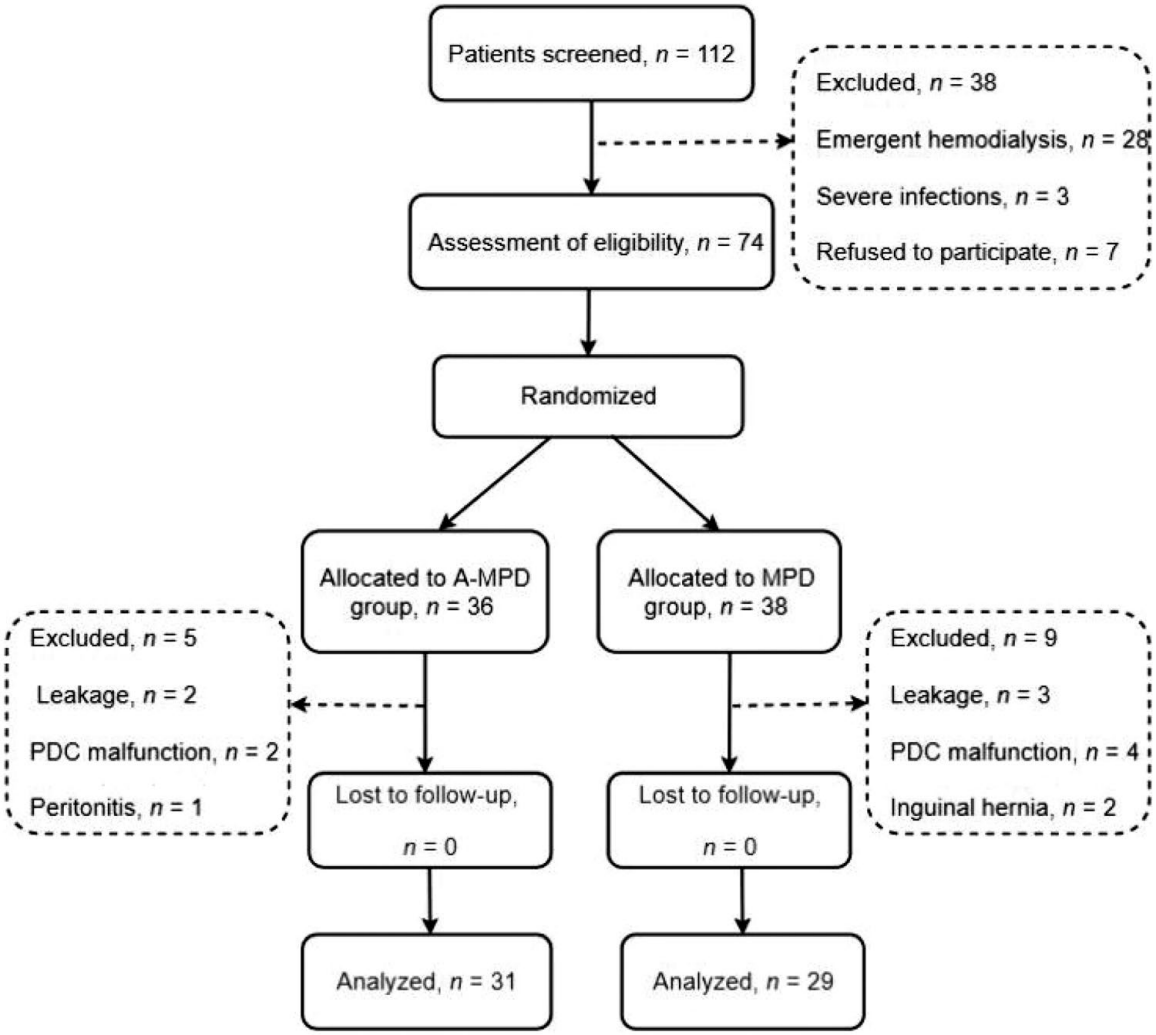
Flow diagram of enrollment, randomization, and follow-up of patients. MPD: manual fluid exchange peritoneal dialysis; A-MPD: combination of automated peritoneal dialysis (APD) with MPD; PDC: peritoneal dialysis catheter.

Overall, 74 patients were enrolled in this study. Among these, 60 completed the study, including 29 and 31 patients in the MPD and A-MPD groups, respectively (Figure 1). The enrolled participants had a mean age of 42.15 ± 12.44 years. As shown in Table 2, the baseline characteristics were similar in both groups regarding age, sex, BMI, cause of ESRD, and laboratory parameters.

**Table 2.** Demographic and baseline characteristics.

,.	A-MPD	MPD	*P* value
Number of patients	36	38	
Male, *n* (%)	21 (58.33%)	22 (57.89%)	0.970
Age (year)	39.58 ± 10.07	44.58 ± 14.04	0.084
Surgery			
Open surgery *n* (%)	24 (66.67%)	31 (81.59%)	0.142
Laparoscopy surgery *n* (%)	12 (33.33%)	7 (18.42%)	0.142
Cause of ESRD *n* (%)			
Diabetic nephropathy *n* (%)	3 (3.23%)	4 (13.79%)	
Hypertensive nephrosclerosis *n* (%)	2 (6.45%)	4 (10.34%)	
Chronic glomerulonephritis *n* (%)	18 (51.61%)	8 (17.24%)	0.139
Others *n* (%)	2 (6.45%)	3 (6.90%)	
Unknown *n* (%)	11 (32.26%)	19 (51.72%)	
Body mass index (kg/m^2^)	22.34 ± 3.28	21.28 ± 3.24	0.169
Body surface area (m^2^)	1.76 ± 0.21	1.72 ± 0.19	0.432
Hemoglobin (g/L)	90.89 ± 17.12	85.87 ± 16.05	0.197
Albumin (g/L)	40.11 ± 4.38	39.21 ± 4.79	0.401
Blood urea nitrogen (mmol/L)	32.21 ± 10.71	29.36 ± 9.52	0.230
Creatinine (μmo/L)	866.44 ± 230.68	827.63 ± 230.81	0.472
eGFR (mL/min/1.73 m^2^)	5.77 ± 1.93	5.83 ± 1.67	0.882
Uric acid (μmo/L)	432.72 ± 139.25	410.53 ± 104.66	0.439
CO_2_-CP (mmol/L)	18.13 ± 3.37	19.33 ± 3.63	0.144
Serum sodium (mmol/L)	139.59 ± 3.00	138.77 ± 3.21	0.262
Serum potassium (mmol/L)	4.63 ± 0.57	4.50 ± 0.73	0.411
Corrected serum calcium (mmol/L)	2.08 ± 0.24	2.07 ± 0.31	0.867
Serum magnesium (mmol/L)	1.02 ± 0.20	0.96 ± 0.24	0.230
Serum phosphorous (mmol/L)	1.93 ± 0.43	1.87 ± 0.57	0.591
iPTH (pmol/L)	35.57 ± 27.84	48.70 ± 33.52	0.095
NT-pro-BNP (ng/L)	6702.76 ± 11395.45	7323.04 ± 10968.84	0.721

eGFR: estimated glomerular filtration rate; CO_2_-CP: carbon dioxide combining power; iPTH: intact parathyroid hormone; NT-pro-BNP: N-terminal pro-brain natriuretic peptide.

Corrected serum calcium = serum calcium(mmol/L)+(40 albumin(g/L))*0.02.

### 3.2. Comparison of the laboratory parameters

After the five-day USPD treatment, the serum levels of creatinine, blood urea nitrogen, albumin, potassium, magnesium, and iPTH decreased dramatically (*p* < 0.05), whereas the corrected serum calcium and CO_2_-CP increased significantly (*p* < 0.05) in both groups (Table 3). The uric acid level in the A-MPD group was significantly lower than before USPD (*p* < 0.05, Table 3). Comparison between the two groups showed that the clearance of creatinine, urea nitrogen, and potassium was more notable in the A-MPD group (*p* < 0.05, Table 4). In addition, the A-MPD mode was more potent in increasing CO_2_-CP than the MPD mode (*p* < 0.05, Table 4). Furthermore, no significant differences were found in the changes in the levels of uric acid, albumin, corrected serum calcium, and iPTH after USPD between the two groups (*p* > 0.05, Table 4).

**Table 3.** Self-comparison of laboratory parameters before and after USPD within groups.

Characteristics	A-MPD			MPD		
	Before	After	*p* Value	Before	After	*p* Value
Hemoglobin (g/L)	91.42 ± 17.13	91.58 ± 15.47	0.936	83.66 ± 15.22	84.52 ± 12.26	0.634
Albumin (g/L)	40.47 ± 4.31	37.63 ± 4.07	0.000*	40.01 ± 4.59	36.92 ± 4.61	0.000*
Blood urea nitrogen (mmol/L)	33.06 ± 10.55	23.63 ± 7.55	0.000*	29.55 ± 8.67	25.07 ± 7.29	0.000*
Creatinine (μmo/L)	894.71 ± 234.13	786.94 ± 214.82	0.000*	810.45 ± 188.95	747.93 ± 180.95	0.000*
Uric acid (μmo/L)	439.13 ± 145.29	377.06 ± 103.82	0.015*	415.48 ± 103.69	397.79 ± 99.58	0.309
CO_2_-CP (mmol/L)	18.20 ± 3.53	23.08 ± 2.95	0.000*	19.19 ± 3.56	21.21 ± 2.91	0.013*
Serum sodium (mmol/L)	139.62 ± 2.91	139.99 ± 3.03	0.459	138.81 ± 3.35	139.58 ± 1.79	0.233
Serum potassium (mmol/L)	4.67 ± 0.59	4.10 ± 0.52	0.000*	4.49 ± 0.67	4.24 ± 0.48	0.023*
Corrected serum calcium (mmol/L)	2.07 ± 0.25	2.21 ± 0.18	0.028*	2.04 ± 0.24	2.16 ± 0.18	0.036*
Serum magnesium (mmol/L)	1.03 ± 0.20	0.93 ± 0.12	0.000*	0.98 ± 0.26	0.91 ± 0.176	0.007*
Serum phosphorous (mmol/L)	1.94 ± 0.45	1.92 ± 0.36	0.812	1.79 ± 0.47	1.85 ± 0.50	0.509
iPTH (pmol/L)	34.31 ± 19.96	23.67 ± 14.38	0.043*	48.91 ± 42.55	33.33 ± 27.33	0.010*
NT-pro-BNP (ng/L)	3425.56 ± 5480.51	4498.22 ± 9411.77	0.503	7178.91 ± 10168.68	4030.82 ± 4575.65	0.109

CO_2_-CP: carbon dioxide combining power; iPTH: intact parathyroid hormone; NT-pro-BNP,:N-terminal pro-brain natriuretic peptide. Corrected serum calcium = serum calcium (mmol/L)+(40 albumin(g/L))*0.02.

**Table 4.** Comparison of the difference of laboratory parameters before and after USPD between the two groups.

Characteristics	A-MPD Δ1	MPD Δ2	*p* Value
Hemoglobin (g/L)	–0.16 ± 11.15	–0.86 ± 9.65	0.796
Albumin (g/L)	2.84 ± 3.25	3.09 ± 3.26	0.766
Blood urea nitrogen (mmol/L)	9.43 ± 8.27	4.48 ± 5.88	0.010*
Creatinine (μmo/L)	107.77 ± 95.84	62.52 ± 54.58	0.030*
Uric acid (μmo/L)	62.07 ± 133.38	17.69 ± 91.96	0.142
CO_2_-CP (mmol/L)	–4.89 ± 4.90	–2.02 ± 4.12	0.018*
Serum sodium (mmol/L)	–0.38 ± 2.80	–0.77 ± 3.41	0.625
Serum potassium (mmol/L)	0.57 ± 0.62	0.25 ± 0.56	0.039*
Corrected serum calcium (mmol/L)	–0.14 ± 0.15	–0.12 ± 0.19	0.600
Serum magnesium (mmol/L)	0.11 ± 0.13	0.08 ± 0.14	0.337
Serum phosphorous (mmol/L)	0.02 ± 0.46	–0.06 ± 0.49	0.511
iPTH (pmol/L)	10.64 ± 9.64	15.59 ± 18.48	0.549
NT-pro-BNP (ng/L)	–1072.67 ± 4589.07	3148.09 ± 5930.94	0.098

CO_2_-CP: carbon dioxide combining power; iPTH: intact parathyroid hormone; NT-pro-BNP: N-terminal pro-brain natriuretic peptide.

Corrected serum calcium = serum calcium(mmol/L)+(40 albumin(g/L))*0.02.

Δ1 and Δ2, the value for the difference before and after USPD (before USPD – 5 days after USPD) in A-MPD group and MPD group, respectively.

### 3.3. Short-term PD-related complications

In total, 21 patients experienced short-term complications during USPD treatment (Table 5), of which 14 quit the study (five in A-MPD and nine in MPD) (Table 6). Leakage occurred in four patients in the A-MPD group and five in the MPD group. After suspension of PD for three days, two patients in A-MPD and three patients in MPD still had leakage. These five patients quit the study but succeeded in starting PD again after a longer suspension. There were three patients in A-MPD and four patients in MPD that had catheter blockage. One patient in the A-MPD group had omental encapsulation and one patient in the MPD group had a catheter displacement. The malfunction of the PD catheter did not need correcting (two in A-MPD and four in MPD) after relaxing the bowels, out-of-bed activity, and IP urokinase use, among others. These seven patients quit the study. They had a replacement of the PD catheter under laparoscopy and restarted PD after the operation. One patient in A-MPD developed peritonitis and quit the study. In addition, two patients acquired inguinal hernias and quit the study. However, the incidence of short-term complications and the number of patients who quit the study were not significantly different between the two groups (*p* > 0.05). For those patients who terminated the study, further treatment would involve replacing the PDC under laparoscopy, suspension of PD for a longer time, or initiating urgent hemodialysis if needed.

**Table 5.** PD-related complications during 5-day USPD treatment.

Complications, *n* (%)	A-MPD	MPD	*p* Value
Total	9 (25.00%)	12 (31.58%)	0.530
Leakage	4 (11.11%)	5 (13.16%)	1.000
PDC malfunction	4 (11.11%)	5 (13.16%)	1.000
Catheter displacement	0 (0.00%)	1 (2.63%)	1.000
Catheter blockage	3 (8.33%)	4 (10.53%)	1.000
Omental encapsulation	1 (2.78%)	0 (0.00%)	0.486
Peritonitis	1 (2.78%)	0 (0.00%)	0.486
Catheter-related infection	0 (0.00%)	0 (0.00%)	–
Inguinal hernia	0 (0.00%)	2 (5.26%)	0.494

PDC: peritoneal dialysis catheter.

**Table 6.** Reasons of quitting the study.

Reasons, *n* (%)	A-MPD	MPD	*p* Value
Total	5 (13.89%)	9 (23.68%)	0.282
Leakage	2 (5.56%)	3 (7.89%)	1.000
PDC malfunction	2 (5.56%)	4 (10.53%)	0.675
Catheter displacement	0 (0.00%)	1 (2.63%)	1.000
Catheter blockage	1 (2.78%)	3 (7.89%)	0.615
Omental encapsulation	1 (2.78%)	0 (0.00%)	0.486
Peritonitis	1 (2.78%)	0 (0.00%)	0.486
Catheter-related infection	0 (0.00%)	0 (0.00%)	–
Inguinal hernia	0 (0.00%)	2 (5.26%)	0.494

PDC: peritoneal dialysis catheter.

### 3.4. Time expenditure of nurses treating PD and patients’ test scores

The mean time expenditure for dialysate exchange by nurses for the A-MPD group was 248.45 ± 6.20 min during the five days (including two manual fluid exchanges and connection and disconnection of cycler per day). The mean time expenditure in the MPD group was 256.86 ± 11.70 min/day (including four manual fluid exchanges per day). The time expenditure of nurses treating PD in the A-MPD group was significantly shorter than that in the MPD group (*p* < 0.05, Table 7).

**Table 7.** The time expenditure of PD nurses and test score of patients.

Variable	A-MPD	MPD	*p* Value
Operation time (minutes)	248.45 ± 6.20	256.86 ± 11.70	0.001*
Score of operation exams	183.63 ± 15.83	167.59 ± 20.32	0.002*

The mean test scores of patients in the A-MPD and MPD groups were 183.63 ± 15.83 and 167.59 ± 20.32, respectively. Notably, patients’ test scores in the A-MPD group were significantly higher than those in the MPD group (*p* < 0.05).

### 3.5. PD technique survival and patient survival

Here, 60 patients were followed up for 6 months. Two patients in the A-MPD group developed peritonitis during follow-up; one underwent removal of the PDC and was switched to hemodialysis, while the other resumed peritoneal dialysis after antibiotic treatment. In contrast, one patient in the MPD group received renal transplantation, and two patients were lost to follow-up. No statistically significant difference in PD technical survival was found between the two groups (*p* > 0.05, Table 8). In addition, no deaths occurred during the follow-up period.

**Table 8.** Technique and patient survival during 6 months.

	A-MPD	MPD	*p* Value
PD technical survival rate (%)	96.77%	89.66%	0.346
Transfer to HD, *n* (%)	1 (3.23%)	0 (0.00%)	1.000
Combined with HD, *n* (%)	0 (0.00%)	0 (0.00%)	–
Transfer to renal transplantation, *n* (%)	0 (0.00%)	1 (3.45%)	0.483
Death *n* (%)	0 (0.00%)	0 (0.00%)	–
Total number of follow-up, *n*	31	29	–
Losing to follow-up, *n* (%)	0 (0.00%)	2 (6.90%)	0.229

## 4. Discussion

This study is the first to combine APD with MPD in the treatment of USPD. Compared with MPD, the A-MPD mode had a better effect on removing small-molecule toxins. Simultaneously, the A-MPD mode could effectively save time for nurses treating PD and improve the skill training of patients. The incidence of short-term complications, PD technique survival, and mortality were not significantly different between the two modes. Therefore, the A-MPD mode may be recommended as an adaptable and suitable PD mode for USPD treatment.

Recent studies have shown that the APD mode has a better effect on removing small-molecule toxins than MPD in USPD [8,9]. Similarly, our study found that the A-MPD mode had better dialysis adequacy for small-molecule toxins than MPD. We considered that the difference was also related to the higher total dialysate volume, the higher number of exchanges, and the longer total dwell time. Therefore, the A-MPD mode has the advantage of adequate dialysis as the APD dose. In addition, A-MPD has more benefits than APD alone. First, A-MPD provides the experience and training of the two PD modes concurrently, thus enabling patients to select the most suitable mode after discharge. Second, A-MPD provides an opportunity for nurses to observe the filling and draining of the dialysate during the two manual exchanges, which may help identify PD-related complications over time.

The design of the A-MPD model should consider the adequacy of dialysis, the practicability, the time expenditure of nurses treating PD, and the tolerance of patients. For the MPD mode, previous studies have adopted four to six exchanges per day [9,11,12]. However, we could only provide four exchanges in the MPD because of the shortage of nurses treating PD. The A-MPD mode provides more dialysate volume, cycles, and retention time, which makes adequate dialysis practicable considering the cost of nurse time. Finally, patient tolerance should be considered. Connecting with the cycler for a long time could improve the removal of toxins and water, although it would also confine the patients to their beds, which might cause flatulence and result in PDC migration. Furthermore, prolonged bed rest may cause patients to resist and refuse to use a cycler. In our study, we set the time for APD to 600 min during nighttime, and no patients in the A-MPD group were reluctant to undergo treatment.

An interesting finding of our study is that patients in the A-MPD group received higher test scores with shorter time expenditures for nurses treating PD. Once the cycler was connected to the PDC, the nurses treating PD did not need to wait by the patient’s bed or weigh the dialysate, which obviously reduces time expenditure. This probably gave the nurses treating PD more time and patience to conduct patient training and promptly correct patients’ mistakes during dialysate exchange. Meanwhile, if the patients only had APD, they learned MPD using only the artificial model. The patients then had no opportunity to do MPD on themselves and had no real practical experience. This finding further demonstrates that A-MPD could reduce the burden of the nurse and improve the skill of patients, which is more suitable than APD or MPD alone.

Previous studies have found that the APD mode could increase the loss of serum albumin more than MPD in patients during USPD [14,15]. A higher PD dose of dialysate may be associated with more loss of albumin [16–19]. In those studies [14,15], the total dialysate volume in the APD group was 6.5–8 L; our study adopted 4.6–7 L. Here, serum albumin levels decreased notably in both groups after the five-day USPD, and the decrease in albumin levels before and after USPD was not significantly different between the two groups. Uremia itself is a risk factor for malnutrition and needs to be rapidly corrected with high PD doses during USPD. Therefore, we consider that a reasonable prescription setting in the A-MPD mode is of great importance, considering both the albumin loss and rapid alleviation of the uremia status.

Dialysate leakage is a common complication after PDC implantation, and its incidence in USPD reported in the literature ranges from 2.2% to 37% [8,12,20,21]. Therefore, to avoid this issue, an appropriately small volume of peritoneal fluid was prescribed according to the patient’s BSA to reduce abdominal pressure and thus reduce the incidence of peritoneal fluid leakage. All patients at our center started USPD within 48 h of intubation with a low PD dialysate infusion volume (700–1000 mL). The incidence of dialysate leakage in the A-MPD group was 11.11%.

No significant difference in the PD technique survival or mortality was found between the two groups during the six-month follow-up period. Therefore, we consider that the five-day USPD treatment would not affect the PD technical survival rate and mortality during follow-up because of our study’s limited sample size and many other factors that affect PD after discharge.

In this study, the PD catheters were implanted by open surgical dissection or laparoscopy. Recently, percutaneous catheterization was more and more widely used in USPD. A real-world, multicenter, retrospective cohort study found percutaneous catheterization was more commonly used for a break interval <24 h and laparoscopic or open surgery method was more commonly used for a break interval >7 days [22]. Another study compared percutaneous with surgical implantation in USPD [23]. It found infectious and mechanical complications showed no significant differences between the two methods and concluded that percutaneous implantation could be applied safely by nephrologists with no break-in period in USPD. Therefore, we think the optimal implantation method of PD catheter in USPD needs to be verified by more studies in the future.

Our study had some limitations. First, it was a single-center study with a limited number of patients. Therefore, more multicenter studies with larger sample sizes are required. Second, the optimal prescriptions for USPD still need to be determined. Here, we considered the dialysis dose, dwell time, exchange cycles, the patient’s tolerance, safety, the patient’s training, and time expenditure. Future studies should consider the symptoms, urine volume, and ultrafiltration, among others, and individualize the prescription according to each patient.

## 5. Conclusion

The combination of APD with MPD during USPD treatment had more advantages in removing small-molecule toxins, correcting hyperkalemia and acidosis, reducing the burden of nurses by saving time and improving patients’ skills in fluid exchange. Therefore, this mode could be recommended as an adoptable and suitable PD modality for USPD in the future.

## Data Availability

The datasets used and analyzed during the current study are available from the corresponding author upon reasonable request.

## Appendix 1. Evaluation and scale of operating procedures

**Table:**

1. Clean and dry the hands; clean the operating board.	5 points
2. Gather the required supplies: the solution bags, face-mask, mini-cap package, clamps, basin, hand-sanitizer, etc.	5 points
3. Wear a face-mask and wash hands with routine steps.	5 points
4. Check the expiration date, solution amount and concentration on the package of the twin-bag fluid. Open the outer package, take out the twin-bag fluid, check for leaks. Make sure the pull ring and pipelines are in place.	10 points
5. Hang the twin-bag fluid, separate the lines, and place the drain bag in the basin on the ground.	5 points
6. Close the filling line and drain line respectively with two clamps.	5 points
7. Expose the transfer set and check the twist clamp is closed.	10 points
8. Remove the pull ring from the twin-bag connector, remove the mini-cap from the transfer set.	10 points
9. Immediately attach the twin-bag connector to the transfer set by twist the twin-bag connector, avoiding crossing over when making connections.	10 points
10. Remove the clamp on the draining line.	10 points
11. Open the twist-clamp on the transfer set a half turn and start to drain, observing flow speed of the drained fluid.	10 points
12. After draining, place the clamp on the draining line and close it. Close the twist clamp on the transfer set.	10 points
13. Open the switch between the fluid and filling line.	10 points
14. Open the clamps on the filling line and the draining line for 5 s, flushing fresh fluids into the drain bag.	10 points
15. Close the clamp on the drainage line.	10 points
16. Open the twist-clamp on the transfer set a half turn to fill.	10 points
17. After filling, close the clamp on the filling line.	10 points
18. Close the twist-clamp on the transfer set.	10 points
19. Check the package of mini cap. Open the package and check whether the cap is infiltrated with iodophor solution.	5 points
20. Check the twist camp on the transfer set is closed	5 points
21. Unscrew the twin-bag connector from the transfer set, keeping the transfer set in the hand.	10 points
22. Apply a new mini-cap to the transfer set immediately, avoiding crossing over.	10 points
23. Check the drain fluid for clarity and color.	5 points
24. Weigh the drain bag and record the ultrafiltration volume.	5 points
25. Dispose the drainage fluids and disposable items.	5 points

The total score is 200 points. The patient is considered to be qualified if he or she got more than 120 points.
